# Interaction of physical activity and interoception in children

**DOI:** 10.3389/fpsyg.2015.00502

**Published:** 2015-04-28

**Authors:** Eleana Georgiou, Ellen Matthias, Susanne Kobel, Sarah Kettner, Jens Dreyhaupt, Jürgen M. Steinacker, Olga Pollatos

**Affiliations:** ^1^Health Psychology, Institute of Psychology and Education, Ulm University, Ulm, Germany; ^2^Research Group “Join the Healthy Boat – Primary School”, Sports and Rehabilitation Medicine, Ulm University, Ulm, Germany; ^3^Institute of Epidemiology and Medical Biometry, Ulm University, Ulm, Germany

**Keywords:** interoceptive sensitivity, physical performance, metabolic equivalent, childhood/youth, interoception, self-regulation

## Abstract

**Background:** Physical activity (PA) is associated with positive health outcomes, whereas physical inactivity is related to an increased risk for various health issues including obesity and cardiovascular diseases. Previous research indicates that interindividual differences in the perception of bodily processes (interoceptive sensitivity, IS) interact with the degree of PA in adults. Whether there is a similar relationship between PA and IS in children has not been investigated yet. Therefore, the aim of this study was to investigate the interaction between IS and PA during physical performance tasks and in everyday situations. **Methods:** IS was assessed using a heartbeat perception task in a sample of 49 children within the health promotion program “Join the Healthy Boat” which is implemented in several primary schools in the southwest of Germany. PA was examined using a physical performance task, assessing the distance covered during a standardized 6-min run. In a subsample of 21 children, everyday PA was measured by a multi-sensor device (Actiheart, CamNtech, Cambridge, UK) during five consecutive days with more than 10 h of daily data collection. **Results:** Children with higher IS performed better in the physical performance task. Additionally, based on energy expenditure defined as metabolic equivalents, IS was positively correlated with the extent of light PA levels in the morning and afternoon. **Conclusion:** Our findings reveal that IS interacts positively with the degree of PA in children supporting the idea that interoception is important for the self-regulation of health-related behavior.

## Introduction

The feedback of afferent signals arising from within the body to the brain and their perception is commonly known as *interoception* (Vaitl, 1996; Cameron, 2001), while the sensitivity in perceiving bodily signals is known as *interoceptive sensitivity* (IS). Referring back to the emotion theories of James (1884) as well as Schachter and Singer (1962), who postulated that there is no emotional experience without the perception of bodily changes, a substantial body of research demonstrates evidence for the suggested close link between interoception and emotional processes in recent decades (for reviews, see Wiens, 2005; Herbert and Pollatos, 2008). Furthermore, the somatic marker theory of Damasio (1994, 1999) emphasized the relevance of visceral and somatosensory feedback from the body to the brain, mapped in distinct higher brain areas, for the emergence of emotions and the regulation of one’s behavior. Thus, Damasio inspired studies that demonstrated the benefit of a person’s high degree of bodily sensitivity to regulate emotional decision making (e.g., Werner et al., 2009; Dunn et al., 2010, 2012) as well as to process emotions (Wiens et al., 2000; Barrett et al., 2004; Pollatos et al., 2005; Herbert et al., 2007a). Furthermore, the feedback of internal bodily signals is related to the self-regulation of physical activity (PA). The self-perception of bodily signals, like breathing or other internal bodily processes based on cardiovascular activity, might influence the amount of physical effort, when undertaking PA and is known as the self-control of physical load (Pennebaker and Lightner, 1980; Herbert et al., 2007b).

Moreover, one study investigated the regulation of PA in relation to interoceptive processes (Herbert et al., 2007b) and highlighted that these two processes might interact positively with each other. In this study, Herbert et al. (2007b) instructed their participants, aged 20–40, to cycle on a bicycle ergometer for a constant period of time (15 min), whilst being free to control their cycling pace. Results showed that good heartbeat perceivers set their physical endeavor to a lesser extent, demonstrating a more enhanced perception of their exhaustion and of their internal bodily states, than poor heartbeat perceivers. Furthermore, good heartbeat perceivers revealed lower changes in their heart rate, stroke volume and cardiac output. This suggested that participants with higher IS where those who at the same time regulated better their physical load in a performance task. Moreover, Herbert et al. (2007b) implemented this study in terms of regulating physical load in a performance task (cycling task). However, it remains a rather open question whether interoceptive processes do also interact with PA in terms of day to day PA (i.e., in terms of PA under free-living conditions). Lastly, in this study there was no evidence found showing that physical fitness does play a role when differentiating the good from the bad heartbeat perceivers.

On the other hand, but in line with this question, two older studies suggested that a higher state of fitness might be advantageous for better IS (Borg and Linderholm, 1967; Montgomery et al., 1984). Montgomery and Jones (1984) also found that the best predictor for IS in men is the body’s fat content. In agreement with these results, Herbert and Pollatos (2014) also demonstrated an inverse relationship between the body mass index (BMI) and IS in adults, and that overweight and obese individuals are less accurate in perceiving bodily signals. These studies suggest that interoceptive processes and individual differences in IS are important for physical fitness or weight status in adults and are of high importance to health-related questions. Although being overweight is established in childhood and youth, there is scant research in examining these questions in children.

Most of the aforementioned studies assess and quantitate IS in terms of people’s ability to perceive their own heartbeat. Assessment of heartbeat perception in adults has been shown to be sufficiently reliable (Schandry, 1981; Mussgay et al., 1999), to have substantial interindividual differences (Ehlers and Breuer, 1992; Domschke et al., 2010) and to correlate with the ability to detect changes in other autonomic innervated organs (Whitehead and Drescher, 1980; Harver et al., 1993; Herbert et al., 2012). One of the most frequently used methods to measure cardiac sensitivity is the *Mental Tracking Method* proposed by Schandry (1981; Dunn et al., 2007; Herbert et al., 2007b; Pollatos et al., 2009; Ainley et al., 2012). A recent study by Koch and Pollatos (2014a) adapted this method for children by shortening the intervals used. In spite of the great importance of interoceptive processes for health processes, only a few studies exist that assess IS in children. To our knowledge, there are only four studies that assess heartbeat perception in children, either using the original Schandry paradigm (three trials with the length of 35, 25, and 45 s; see Eley et al., 2004, 2007; Schmitz et al., 2012) or the adapted version (Koch and Pollatos, 2014a,b). The internal consistency of the shorter adapted version was found to be excellent (Cronbach’s α = 0.91) in a large sample of about 1350 children aged between 6 and 11 years (Koch and Pollatos, 2014a). Additionally, Koch and Pollatos (2014a) investigated the distribution of cardiac sensitivity in relation to general emotional processing and found correlations to interpersonal emotional intelligence and adaptability. Relevant findings were the associations between interoceptive processes and anxiety symptoms (panic and social anxiety) in small samples of eight to 12-year-old children (Eley et al., 2004, 2007; Schmitz et al., 2012). Furthermore, cardiac sensitivity in children can be seen as a dynamic developmental process, that has weaker but yet similar characteristics and relations to emotional parameters than found in adults (Koch and Pollatos, 2014a).

The scope of the present study was to investigate the interrelation between PA in a performance task and in day to day activities (under free-living conditions) with IS in a main sample of 49 primary school children and moreover in a subsample of 21 in the context of the “Baden Württemberg Study,” which evaluated the effectiveness of the health promotion program “Join the Healthy Boat” in Southwestern Germany. Taking into account the majority of the studies regarding this matter in adults (Borg and Linderholm, 1967; Montgomery et al., 1984; Herbert and Pollatos, 2014) and the lack of studies in this field among children, we hypothesized that a healthy and fit physical state is associated with higher IS in children. This should be reflected in a better performance when participating in a physical task as well as in a higher level of daily PA in children. Furthermore, considering the results of the study from Herbert et al. (2007b), we further hypothesized that children with a higher IS will also demonstrate a finer ability to regulate their PA, in comparison to children with low IS.

## Materials and Methods

### Participants

Participants were children of the third wave of a larger on-going intervention study (“Join the Healthy Boat—Primary School”, Schreiber et al., 2014). Our data derived from a subsample of the Baden-Württemberg Study, which evaluated the health promotion program “Join the Healthy Boat—Primary School” in the south-west of Germany. Protocol and study design of the Baden-Württemberg Study have been depicted elsewhere (Dreyhaupt et al., 2012). In total, 1047 children in the age of 9.59 (SD = 0.63) years from third to fourth grade were recruited from primary schools in the federal state of Baden-Württemberg, after legal guardians had provided written informed consent. Approval for the study was obtained from the Ministry of Education as well as from the University’s Ethics Committee.

For logistical reasons (scope of measurements of the Baden-Württemberg study and distances between schools) objective PA assessments and IS measurements were only carried out in different schools in the region of Ulm and among fewer children. The sample used in the present analyses consisted of 49 children and the subsample of 21 respectively with complete data on all variables of interest. Children were tested individually with regard to IS in a separate room at school. Additionally, all participants filled out questionnaires regarding their health status, in order to fulfill all criteria for participating in this study. In total, there were 49 healthy participants, 20 girls (40.8%) and 29 boys (59.2%) with a mean age of *M* = 9.72 years (SD = 0.58) in the main sample. In the subsample of the study, there were 21 children in total, 10 boys (47.6%) and 11 girls (52.4%) with a mean age of *M* = 9.97 years (SD = 0.39).

### Performance Task for Physical Fitness

For the measurement of children’s physical fitness, the 6 min run-performance task was conducted. The Dordel-Koch-Test (Dordel and Koch, 2004; Graf et al., 2004) is conducted in order to measure the developmental state of the participant’s basic motoric skills in accordance to intensity, coordination and endurance of the movement. The 6 min run is a subtask of the Dordel-Koch-Test, which assesses a person’s aerobic endurance. It could be compared to a screening test, in which cardiopulmonary stamina is being measured. The participants of this task had to run around a volleyball field (round length 54 m) for 6 min. The last round was announced by the instructor of the task, who, afterward, marked the rounds already covered by the children. For the evaluation of this task, the aggregation of the number of completely covered rounds and the distance of the last round were calculated.

The 6-min-run task is a highly standardized assessment tool, which is frequently used not only because it is extremely time-saving, but also relatively simple to conduct and unambiguous. For this reason, it has a high level of objectivity. As far as the norm values are concerned, the reliability and objectivity are *r* = 0.91 for the total sample. Von Haaren et al. (2008) also reported reliability values that swayed between *r* = 0.61 and 0.92. Moreover, they compared the results regarding the VO_2max_ (among others an essential indicator for the aerobic stamina) between the 6-min-run performance and a treadmill test and they noticed a strong connection between those two performance tasks (*r* = 0.69).

### Measurement of Daily Physical Activity

Within the context of the Baden-Württemberg study, daily PA was measured in a subsample of 21 primary school children, in order to examine if current PA recommendations of at least 60 min MVPA (moderate to vigorous PA) per day were met, not only during school time, but also during spare time. After the informed consent of the parents, PA was measured via a multi-sensor device (Actiheart^®^, CamNtech Ltd., Cambridge, UK).

The Actiheart^®^ is a light multi-sensor device that combines recordings of the accelerometer (in counts per minute) and of heart rate (in beats per minute). This multi-sensor device was attached using two standard electrocardiograph electrode pads on the children’s chest. The children were instructed to follow their daily routine while wearing the Actiheart^®^ and not to remove the device even during sleep for five consecutive days (á 24 h). Energy expenditure based on metabolic equivalents (METs) was calculated by the Actiheart^®^ software using a branched model approach, previously validated in children (Corder et al., 2007). For this specific recording, a 15-s epochs recording interval was selected. Besides heart rate and accelerometry data, gender, age, body weight, and height were also taken into account in order to assess MET levels. MET was divided into three categories: sedentary (<1.5 METs), light (1.5–3.0 METs), moderate to vigorous (>3.0–6.0 METs) (Pate et al., 1995).

For data assessment, some specific criteria had to be fulfilled in order for the data to be included in the analysis. Only recordings with at least 10 h of daily data collection were included in this sample. Moreover, recordings of 5 days in total were selected, in which at least 1 day was a weekend day and the rest were weekdays, so as to secure a high reliability among children (see also Trost et al., 2000). All recordings between 11 pm and 6 am were removed as this time was considered as normal sleeping time. Additionally, recordings that included more than 45 min of sedentary activity, between 9 pm and 11 pm, were also extracted. Furthermore, recording periods of 100 min or more with zero activity counts were also removed. Hesketh et al. (2014) suggested three possible day-segmentations that might correspond to the daily program of the preschool and school children in the UK. We chose a similar day-segmentation, because of the resemblance to the German primary school system. Therefore, each day was divided in three periods, as follows: morning (6 am–12 pm), afternoon (12–5 pm) and, lastly, evening (5–11 pm).

### Heartbeat Perception Task

For the heartbeat perception task, cardiac activity was recorded using the mobile heart frequency monitor RS800CX (Polar Electro Oy, Kempele, Finland). Polar watches are mobile devices that enable easy, non-invasive recording of inter-beat-intervals and were used to assess IS in other studies with children (Koch and Pollatos, 2014a,b). Validity and reliability compared to alternative ECG measurement devices have been proven in children and adults (Radespiel-Tröger et al., 2003; Kingsley et al., 2005; Gamelin et al., 2008; Nunan et al., 2008). Due to the fact that the study took place in the school setting, the strap with the electrodes was attached to both hands and secured on a table. Signals were sampled at 1000 Hz and analyzed by the corresponding Polar ProTrainer five software (version 5.40.172).

The heartbeat perception task was performed following the Mental Tracking Method, proposed by Schandry (1981) and identical to the children’s version implemented by Koch and Pollatos (2014a). After a short practice interval of about 10 s, there were three intervals of 15, 20, and 18 s, separated by two standard resting periods of 20 s. During each interval, participants were precisely instructed to silently count their own heartbeats by concentrating on their heart activity while not being allowed to check their pulse or to attempt any other physical manipulations (e.g., holding their breath) that could facilitate the detection of heartbeats. Participants were seated and were given no information as to the length of the intervals or their performance. The test supervisor signaled the beginning and the end of the counting phases by announcing “start” and “stop.” Participants were asked to verbally report the number of counted heartbeats straight after the “stop” signal. IS was determined via the heartbeat perception score, which represents the mean score across the three intervals and which is calculated according to the following transformation:

1/3Σ[1−(|recorded heartbeats−counted heartbeats| /recorded heartbeats)]

Higher scores indicate higher IS, so that the maximum score of 1 indicates absolute accuracy of heartbeat perception.

### Body Mass Index

Children’s body weight and height were taken according to the International Society for the Advancement of Kinanthropy (ISAK; Stewart et al., 2011), with children wearing only underwear and no shoes. More specifically, body weight was measured to the nearest 0.05 kg, using calibrated electronic scales (Seca 862, weighing and measurement systems, Hamburg, Germany). Using a stadiometer, height was measured to the nearest 0.1 cm (Seca 213, weighing and measurement systems, Hamburg, Germany). BMI was calculated (kg/m^2^) and converted to BMI percentiles (BMIPCT) based on national reference data for German children (Kromeyer-Hauschild et al., 2001).

### Statistical Analyses

Continuous variables were summarized as mean and standard deviation; frequencies were used to analyze nominal and ordinal variables. Continuous variables were compared using the two-sample *t*-test and analysis of variance (ANOVA). Regression analyses as well as Pearson correlations and partial correlations were conducted to investigate the relationship between IS, age and gender, BMI and the activity measures.

As far as physical performance is concerned, one hierarchical regression analysis (linear mixed effects regression model, forward stepping) was carried out with IS, BMI and the interaction term IS × BMI as predictors and performed distance as criterion.

As far as everyday PA is concerned, we calculated partial correlations between mean activity at different activity levels and while controlling for age and BMI.

All statistical analyses were conducted using Statistical Package for Social Sciences (SPSS, version 21). Because of the explorative nature of this study, no adjustment for multiple testing was made (Altman, 1991; Victor et al., 2010). A *p* value less than 0.05 was considered significant. The results of all statistical tests are interpreted in an exploratory sense.

## Results

### Sample Descriptives

Sample characteristics on relevant variables are shown in Table 1.

**TABLE 1:** **Characteristics of the main and subsample**.

	*N*^1^ = 49	*N*^2^ = 21
Female, n (%)	20 (40.8)	11 (52.4)
Age (years)	9.72 (0.56)	9.97 (0.39)
BMI (kg/m^2^)	17.33 (2.57)	16.59 (2.26)
BMIPCT	48.04 (27.58)	44.19 (25.89)
Heartbeat perception score	0.59 (0.18)	0.56 (0.16)

All values are mean (SD) unless stated otherwise; N^1^, main sample; N^2^, sub-sample; BMI, body mass index; BMIPCT, BMI percentiles; SD, standard deviation.

### Interoceptive Sensitivity as Assessed by Heartbeat Perception

The mean heartbeat perception score for the main sample was 0.59 (SD = 0.18). Moreover, there was a minimum score of 0.16 and a maximum of 0.91 regarding the heartbeat perception in the overall sample. Taking into account the possible interrelation between gender and heartbeat perception, a two-sample sample *t*-test was conducted, resulting in boys (*M* = 0.62, SD = 0.19) and girls (*M* = 0.55, SD = 0.17) not differing in their IS [*t*(46) = 1.2, *p* = 0.24]. The same finding occured after comparing gender and heartbeat perception in the subsample [*t*(19) = 1.9, *p* = 0.07]. No significant gender difference was found after considering BMIPCT as a covariate. Accordingly, a one way ANOVA was conducted in order to examine heartbeat perception and age, after dividing age in three categories. No differences were found between these variables [*F*(2,45) = 0.46, *p* = 0.63] in the main sample and in the subsample, respectively [*F*(1,19) = 0.07, *p* = 0.79].

### Physical Fitness

In the physical performance task, boys covered a mean distance of 1003.6 meters (SD = 138.5), whereas girls’ mean covered distance was 904.97 meters (SD = 131.77). Moreover, boys and girls showed significantly different physical performances in the 6-min-run [*t*(45) = 2.45, *p* = 0.02, *d* = 0.73]. The distance covered in 6 min were classified, according to Jouck (2008) in different grades from 1 to 6, taking into account not only the gender but also the age. Mark one refers to an excellent physical performance and mark six to a poor physical performance. As far as minimum and maximum marks of the participants go, there was a deviation ranging from 2 to 6. On the subject of differentiating the physically fit toward physically non-fit children, a dichotomisation of the median value was implemented, resulting in physically fit children having either 2 or 3 as a mark, in comparison to non-physically fit children, who gained the marks 4, 5, or 6. Finally, 31 participants in this study were classified as physically fit, and 18 as physically non-fit. The data showed no significant difference between the different age groups and physical fitness of the main sample [*F*(2,44) = 1.32, *p* = 0.27].

### Relationship Between IS and Physical Fitness

**FIGURE 1:**
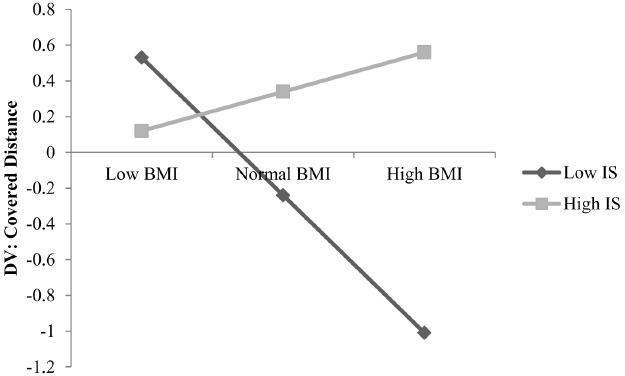
**Interaction between IS (low and high), covered distance and body mass index (BMI); Low BMI, normal BMI, high BMI: based on the national reference data for German children (Kromeyer-Hauschild et al., 2001); DV: dependent variable; classification of low and high IS according to the method of median split**.

In order to examine the relationship between IS and physical fitness, we conducted a linear mixed effects regression analysis (forward stepping) with the covered distance of the 6 min run-performance task as criterion, and BMI, IS and the interaction term of BMI × IS as predictors. The criterion was explained by BMI (*T* = –2.07, β = –0.28, *p* = 0.04), IS (*T* = 2.02, β = 0.29, *p* = 0.04) and the interaction between both [*T* = 2.14, β = 0.32, *p* = 0.04; *F*(3,45) = 5.09, *p* = 0.00, *R* = 0.50, *R^2^* = 0.25]. These effects are depicted in Figure 1.

More specifically, a higher IS is associated with a greater covered distance which implies a higher physical fitness. Moreover, a higher physical fitness is positively associated with the interaction between IS and BMI. Therefore, Figure 1 depicts a positive interaction between high IS, covered distance and BMI, but a strong negative interrelation between low IS, covered distance and BMI. In other words, Figure 1 reveals a positive correlation between physical fitness and BMI among good heartbeat perceivers and a negative correlation between physical fitness and BMI among bad heartbeat perceivers.

### Daily Physical Activity

In total, 21 children, forming the subsample of the study, had valid PA data for five consecutive days with a mean daily wear time of 846 min (corresponding to about 14 h, *M* = 846.2 min, SD = 207.1; see Table 2).

**TABLE 2:** **Children’s average daily physical activity levels in minutes segmented across the day**.

	Daily total (min/day) (SD)	Morning (min/day) (SD)	Afternoon (min/day) (SD)	Evening (min/day) (SD)
Recorded time	846.2 (207.1)	299.5 (75.8)	250.7 (82.8)	296.0 (85.0)
Sedentary PA	156.1 (70.4)	58.2 (77.6)	20.6 (29.9)	77.3 (81.8)
Active PA	690.1 (203.9)	241.3 (117.2)	230.1 (116.6)	218.7 (110.2)
Light PA	636.6 (187.9)	217.2 (87.9)	213 (81.2)	206.4 (67.7)
MVPA	53.5 (70.6)	24.1 (29.9)	17.1 (33.6)	12.3 (22.4)

All values depicted are in minutes/day; in parentheses: SD, standard deviation; PA, physical activity; Active PA, sum of light PA and MVPA; MVPA, moderate to vigorous physical activity; morning: 6 am–12 pm; afternoon: 12–5 pm; evening: 5–11 pm.

Boys (*M* = 237.5, SD = 61.02) and girls (*M* = 178.2, SD = 63.10) differ as far as their light PA in the evening is concerned [*t*(19) = 2.18, *p* = 0.04, *d* = 0.95]. Moreover, the data showed a difference regarding sedentary activity [*t*(19) = 1.27, *p* = 0.02, *d* = 1.1], with girls (*M* = 115.04, SD = 88.15) being more sedentary in the evening in comparison to boys (*M* = 35.8, SD = 50.7). Concerning MVPA there was also a gender-specific difference [*t*(19) = 3.9, *p* = 0.01, *d* = 1.7]. MVPA in the morning was higher for boys (*M* = 44.6, SD = 30.2) in comparison to girls (*M* = 5.5, SD = 12.6). Concerning age, there was no significant difference between the three age groups and the different levels of PA.

### Relationship Between IS and Daily Physical Activity

**FIGURE 2:**
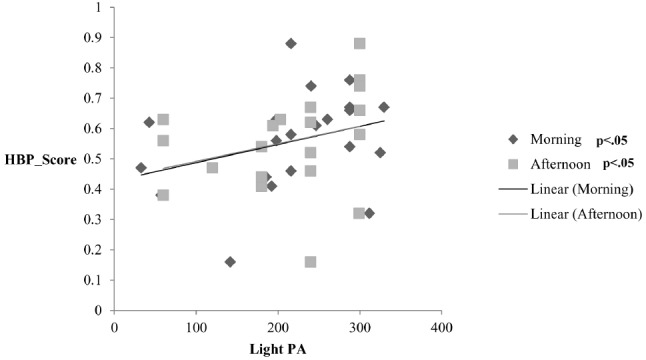
**Partial correlations between interoceptive Sensitivity (IS) and physical activity (PA) in the morning and in the afternoon, after controlling for body mass index (BMI) and age; HBP_Score: heartbeat perception score**.

Examining the relationship between IS and daily PA, we conducted partial correlations after setting BMI and children’s age as control variables. There was a statistically significant correlation between IS and light PA, more specifically in the morning (*r* = 0.39, *p* = 0.04) and in the afternoon (*r* = 0.39, *p* = 0.04), showing that higher IS was positively related to more light PA in the morning and afternoon hours. This interrelation is depicted in Figure 2.

## Discussion

The present study investigated the interrelation between IS and PA among primary school children. Our results demonstrate, firstly, that IS is a determinable measure in children, indicating that primary school children differ considerably in their ability to perceive ongoing signals stemming from the heart. This is in line with recent data from a large representative sample of about 1350 children (Koch and Pollatos, 2014a). Secondly, further findings of this study highlight that physical fitness and IS are positively associated, showing that higher IS is related to a greater distance covered in the 6 min running performance task. These findings are not in line with previous studies regarding IS and physical fitness among adults, for example the study from Herbert et al. (2007b), where good and poor heartbeat perceivers did not differentiate in their fitness level.

Furthermore, results concerning physical fitness and IS revealed also the role of the BMI in this motif. More specifically, these findings indicate that children with a normal BMI and a high physical fitness (according to the 6-min-run) showed a higher IS than those children with a normal BMI but a lower fitness state. Lastly, children with a high BMI and high physical fitness indicated also, surprisingly, a higher IS than those children with a high BMI and a lower physical fitness. Thus, good heartbeat perceivers were these children with a normal to high BMI and with a greater fitness state. These findings could be explained by the fact that BMI could play a role in the degree of IS but the most important factor is one’s physical state. In other words, IS does not seem to decline when a child has a high BMI but also at the same time rather good physical fitness. This is in accordance with a few older studies in adults reporting that a higher state of fitness is advantageous for better IS (Borg and Linderholm, 1967; Montgomery et al., 1984). In contrast, children with a lower physical fitness but with higher BMI seemed, in this study, to be bad heartbeat perceivers. In respect to the possible limitations regarding this finding, the use of BMI instead of BMIPCT when referring to children and the absence of use of alternative anthropometric measurements, such as skin-fold thickness or body girth etc. (Chen et al., 2002), should not be neglected.

Moreover, our daily PA related outcomes reveal a positive association between IS and light daily PA in the morning as well as in the afternoon. No significant correlations were observed with the moderate to vigorous activity level, though. This result could be partly due to the fact that our sample was quite small and only 6 (28.6%) participants of the total sample met current PA recommendations of at least 60 min MVPA per day. Referring to former research, there are substantial methodological differences whether daily activity was assessed by self-report or by the use of objective measurements like the Actiheart device in the present study, and which cut-off points were used when determining MVPA (see Borracino et al., 2009; Aznar et al., 2011; Ekelund et al., 2011; Kettner et al., 2013). Being sensitive to one’s bodily signals might constitute a positive precondition for effective self-regulation of behavior, as it was suggested for the field of emotion regulation (see Füstös et al., 2013). Taking both obtained results together, we can show that both in a performance task as well as in day to day life, IS interacts positively with PA suggesting that the ability to accurately perceive bodily signals is crucially associated with more fitness and daily activity in young children. Therefore, a link between IS and other health-related outcome variables is to be assumed, such as demonstrated in adults (Herbert and Pollatos, 2014) or in children (Koch and Pollatos, 2014a,b).

The fact that we observed positive correlations between IS, physical fitness and light daily PA generates further questions regarding the regulation of PA. A former study by Herbert et al. (2007b) demonstrated that when participants were instructed to cycle on a bicycle ergometer at a speed they felt comfortable with, good heartbeat perceivers covered a shorter distance as compared to poor heartbeat perceivers. The good heartbeat perceivers indicated a more self-controled physical workload, by perceiving better their internal bodily signals and regulating their fatigue. In contrast to the instruction given by Herbert and co-workers, the 6-min performance task used in this study focused on how fit children were with the clear instruction to run as fast and as far as possible in a certain time. The instruction when undertaking everyday PA was to keep on undertaking normal activity as usual, which implied that the participant was not in a situation where his/her performance was being evaluated. Therefore, we hypothesize that higher PA might favor the development of a better ability to identify internal body signals as assessed by IS. The exact developmental mechanisms of interoception remain yet unclear, due to the lack of prospective studies and in general studies concerning the distribution of cardiac sensitivity in children (Koch and Pollatos, 2014a). Future studies should focus on this research gap and assess PA and IS in a longitudinal fashion.

In accordance to former research, we found some gender differences in daily activity between boys and girls (Riddoch et al., 2004; Jago et al., 2005; Borracino et al., 2009). Moreover, in examining physical fitness, our results suggest that boys are more physically fit than girls. This can be explained by the physiology of the male body and more specifically, by the fact that males have a greater muscle mass than females, which implies a greater ability of achieving high levels of PA. This finding is in the same line with the study of Li et al. (2007), who concluded that males covered a greater 6 min walk distance than females in the performance task. Moving on to age and PA, our results reveal that 9–11-year-old participants undertook the same amount of PA. These results are in line with other studies, which suggest that children under the age of 13 indicate the same levels of PA (Strauss et al., 2001). We did not find any difference in IS according to gender, like other studies with adults (Katkin et al., 1981; Jones et al., 1984; Jones, 1995), while Koch and Pollatos (2014a,b) showed a small but significant difference with higher IS in boys. We assume that the sample size is small to show such an effect.

To sum up, our study demonstrated that IS is not only determinable but also diverse among primary school children, emphasizing the fact that IS is based on individual differences and age. Further studies could shed light on the developmentary processes of IS through the life span. Taking into account the lack of studies in this field, in the present study we tried to scrutinize the role of PA and IS in children and our findings demonstrate the first evidence regarding the interaction between physical fitness and IS in young children. Further research is necessary to examine the specific role of BMI as well as the direction of the observed interaction, e.g., by training physical fitness in children and assessing concomitant interoceptive processes, and by using alternative anthropometric measurements apart from BMI (such as skin-fold thickness etc.). In specific, we demonstrated that physical fitness could contribute to a higher IS and in its turn, IS might be trained using PA (Schandry and Weitkunat, 1990). We assume that improving children’s perception of their body signals could contribute to a more effective way of regulating health-related behavior, e.g., by a finer ability to tune their physical load in everyday situations and to prevent exhaustion more effectively. Our results suggest that IS constitutes an important factor associated with PA during childhood. This issue deserves further exploration, by researching a larger sample of children, thanks to the great significance of IS and PA over the life span.

### Conflict of Interest Statement

The authors declare that the research was conducted in the absence of any commercial or financial relationships that could be construed as a potential conflict of interest.
